# Role of Double-Strand Break End-Tethering during Gene Conversion in *Saccharomyces cerevisiae*

**DOI:** 10.1371/journal.pgen.1005976

**Published:** 2016-04-13

**Authors:** Suvi Jain, Neal Sugawara, James E. Haber

**Affiliations:** Department of Biology and Rosenstiel Medical Center, Brandeis University, Waltham, Massachusetts, United States of America; University of California, Davis, UNITED STATES

## Abstract

Correct repair of DNA double-strand breaks (DSBs) is critical for maintaining genome stability. Whereas gene conversion (GC)-mediated repair is mostly error-free, repair by break-induced replication (BIR) is associated with non-reciprocal translocations and loss of heterozygosity. We have previously shown that a Recombination Execution Checkpoint (REC) mediates this competition by preventing the BIR pathway from acting on DSBs that can be repaired by GC. Here, we asked if the REC can also determine whether the ends that are engaged in a GC-compatible configuration belong to the same break, since repair involving ends from different breaks will produce potentially deleterious translocations. We report that the kinetics of repair are markedly delayed when the two DSB ends that participate in GC belong to different DSBs (termed Trans) compared to the case when both DSB ends come from the same break (Cis). However, repair in Trans still occurs by GC rather than BIR, and the overall efficiency of repair is comparable. Hence, the REC is not sensitive to the “origin” of the DSB ends. When the homologous ends for GC are in Trans, the delay in repair appears to reflect their tethering to sequences on the other side of the DSB that themselves recombine with other genomic locations with which they share sequence homology. These data support previous observations that the two ends of a DSB are usually tethered to each other and that this tethering facilitates both ends encountering the same donor sequence. We also found that the presence of homeologous/repetitive sequences in the vicinity of a DSB can distract the DSB end from finding its *bona fide* homologous donor, and that inhibition of GC by such homeologous sequences is markedly increased upon deleting Sgs1 but not Msh6.

## Author Summary

In budding yeast, DNA double-strand breaks (DSBs) are mostly repaired by gene conversion (GC) in which an intact region with homology to both ends serves as template for repair. If only one of the DSB ends shares homology with another region, repair proceeds via break-induced replication (BIR), which, unlike GC, can lead to nonreciprocal translocations. We previously showed that a Recombination Execution Checkpoint (REC) delays BIR thereby preventing it from acting on breaks that can be repaired by GC. Here, we induced two DSBs such that only one end from each break shares homology with an ectopic donor so that although repair can proceed via GC, it will result in DNA rearrangement. We asked whether REC would inhibit GC-mediated repair in this scenario. We found that REC does not restrict such repair; however, repair by GC is faster when both ends are derived from a single DSB. We suggest that the DSB ends engage in a coordinated search for homology that favors both ends engaging the same donor, thus avoiding complex chromosomal rearrangements. Moreover, when the two ends of a DSB share homology with different loci, recombination of one end with its partner impedes repair of the other end.

## Introduction

Correct and efficient repair of DNA double-strand breaks (DSBs) is crucial for cell survival. DSBs can be repaired either by nonhomologous end joining (NHEJ), which involves simple re-ligation of the broken ends with little or no homology, or by homologous recombination (HR) in which an intact homologous sequence serves as template for repair [1–3]. Of all repair pathways, gene conversion (GC) is most extensively used to repair DSBs in *Saccharomyces cerevisiae* [4,5]. GC is a homology-driven process in which the DSB ends are resected to produce 3’-ended single-stranded DNA tails, which become coated with the Rad51 recombinase protein. Rad51 nucleoprotein filaments search for and strand-invade homologous sequences, which then serve as the template for synthesis of a short patch of DNA required to seal the break. During GC, new DNA synthesis is detected within ~30 minutes of strand-invasion, as measured by a PCR-based primer extension assay that requires only ~45 nucleotides to be added to the synapsed 3’ DSB end [6,7]. Repair by GC does not require components of the lagging strand DNA synthesis machinery such as Polα and primase [8]. Even though GC is associated with an elevated rate of mutations, it is the least error-prone mode of DSB repair [9–11]. However, repair can proceed via GC only if both ends of the break share homology with the donor.

If homology to only one of the ends is present, repair proceeds via a different pathway called break-induced replication (BIR) [12–17]. Although BIR involves both leading- and lagging-strand synthesis it does not proceed by a classical replication fork. Strand-invasion by the homologous end leads to the establishment of a migrating D-loop that can copy all sequences distal to the site of homology, while the DSB end that lacks homology is lost by degradation [18–20]. Compared to GC, the efficiency of BIR is quite variable, ranging from ~10% to as much as >90%, depending upon the position of the DSB and its homologous partner, the length of homology and the extent of DNA synthesis required to complete repair [13–15,18]. BIR is kinetically slower than simple gene conversion, and new DNA synthesis, as assessed by the primer-extension assay, does not initiate until ~3 h after Rad51-mediated strand-invasion of the donor sequence by the homologous end in cycling cells [13–15]. Given that the primer-extension assay requires synthesis of at least ~45 nucleotides to yield a PCR signal, it remains possible that shorter stretches of DNA may be added to the synapsed 3’ end during this 3-h period. BIR requires all three major DNA polymerases and associated DNA replication factors such as Cdc7, Cdt1 and the Cdc45-GINS-MCM helicase complex [14,21] although the MCM helicase was found to be less important when a DSB end shares extensive homology with a donor, such as a homologous chromosome [20]. Only components specifically needed for origin-dependent DNA replication (Cdc6 and the ORC proteins) appear to be completely dispensable for BIR. In addition, BIR requires the 5’ to 3’ helicase, Pif1 [19,20], as well as Pol32 [13,14], the nonessential subunit of DNA polymerase δ [22]. Furthermore, BIR is blocked by mutations in the PCNA replication clamp protein that have little effect on replication or gene conversion [21].

When a DSB could be repaired by either GC or BIR, the GC outcomes prevail [15]. We have previously shown that a Recombination Execution Checkpoint (REC) mediates the choice between the GC and BIR pathways prior to the actual initiation of stable repair synthesis [13]. This choice is based on the topology of the engaged ends such that even when homology to both ends is present, if the homologies lie far from each other or if they are close together but in the wrong orientation, REC restricts the rapid initiation of new DNA synthesis from the synapsed ends. In the absence of such a restriction, both ends could initiate independent/uncoordinated BIR-like repair events, some of which may get resolved by single-strand annealing to produce a gap-repair GC outcome; but in many instances repair would yield a nonreciprocal translocation. If a DSB occurred within a repeated sequence, the two ends could engage two different templates and their uncoordinated repair could produce two non-reciprocal translocations. Therefore, REC may play an important role in maintaining genome integrity and preventing chromosomal translocations by imposing a delay in the initiation of BIR-mediated repair, presumably providing more time for the DSB ends to find homology in the vicinity of each other, while ensuring quick and efficient repair when ends are engaged in a GC-compatible configuration. This delay in the initiation of BIR is partially suppressed by deletion of the Sgs1 helicase [13]. Recently we have shown that the kinetics of BIR becomes as rapid as repair of a short 1.2 kb gap when both Sgs1 and Mph1 helicases are deleted [23].

Since REC is able to sense how the DSB ends are engaged in terms of their orientation and distance with respect to each other [13], we wished to examine if it can also sense the origin of the two ends. In other words, would REC impede repair if ends from two different breaks were involved in repair, even if the two ends could synapse close to each other at the donor in the correct orientation? It is plausible that impeding such repair would restrict deleterious chromosomal translocations that can arise from repair of ends belonging to two different breaks. Besides REC, DSB end-tethering may also play a role in preventing such chromosomal translocations. It has been shown that the ends of a DSB remain tethered to each other for up to several hours after the induction of a break [24–27]. This end-tethering is dependent on several factors including the Mre11-Rad50-Xrs2 complex (MRX), Tel1, Sae2, γ-H2AX and Rad52 such that deletion of either of these factors results in un-tethering of the DSB ends in ~10–25% of the cells. End-tethering has been shown to play an important role in preventing NHEJ-mediated chromosomal translocations, presumably by limiting the interaction between ends from different breaks. However, the importance of DSB end-tethering in HR-mediated repair has not been carefully examined. To address these questions, we compared the efficiency and kinetics of repair between strains in which the DSB ends involved in repair either arose from the same break (Cis) and were tethered together or originated from two different breaks (Trans) and therefore, were not tethered to each other. We found that REC cannot sense the origin of the engaged ends *per se* and does not play a role in restricting GC-mediated repair in Trans. Nevertheless, the kinetics of repair in Trans are much slower relative to repair in Cis; however, this delay is due to the involvement of the opposite end of one of the DSBs in another, competing repair event. We suggest that DSB ends often remain tethered and travel together during the homology search, such that if one end engages with its homologous partner, it influences the fate of the other end by dragging it along.

In the course of this work we also found that neighboring sequences can seriously affect the efficiency of homologous recombination. We observed a sequence-context related diminution in repair both during a gene conversion event as well as during single-strand annealing.

## Results

**Fig 1:**
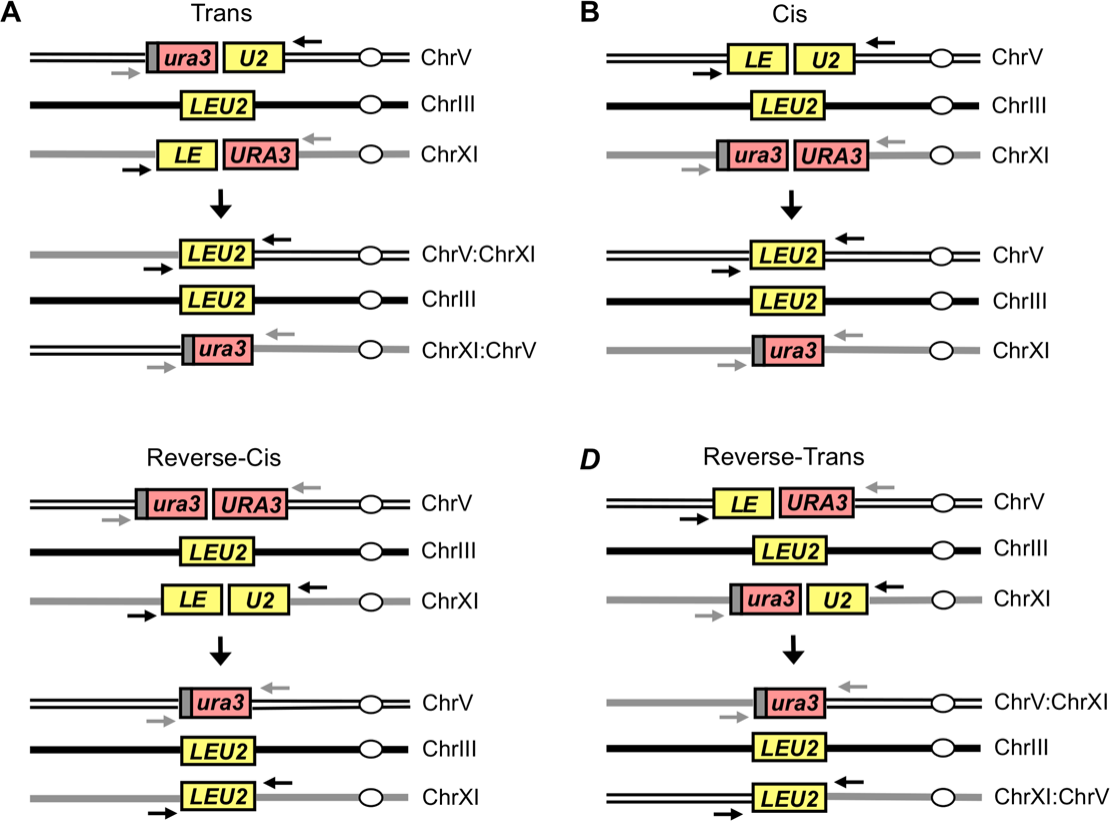
Schematic representation of the strains used to study repair in different configurations. (A) Schematic of the strain used to study repair in Trans (strain YSJ379). A 5’ truncated *ura3-HOcs-U2* cassette is present at the *can1* locus on the left arm of Chr V, a *LEU2* donor is present distal to the *YCL048W-A* locus on the left arm of Chr III and an *LE-HOcs-URA3* cassette is present distal to the *BUD2* locus on the left arm of Chr XI. Bottom panel indicates the repair outcome in which the *LE* end from the break on Chr XI and the *U2* end from the break on Chr V are repaired by GC (using the *LEU2* donor on Chr III), and the *ura3* end from the break on Chr V and the *URA3* end from the break on Chr XI are repaired by interchromosomal SSA resulting in deletion of one of the *URA3* repeats. Both repair events in the Trans strain are associated with a reciprocal translocation between chromosomes V and XI. (B) Schematic of the strain used to study repair in Cis (YSJ357). Bottom panel indicates the repair outcome in which the HO break within *leu2* on Chr V is repaired by GC (using the *LEU2* donor on Chr III), and the HO break on Chr XI is repaired by intrachromosomal SSA resulting in deletion of one of the *URA3* repeats. (C) Schematic of the Reverse-Cis strain (tNS2614) in which the positions of the *LE-HOcs-U2* and *ura3-HOcs-URA3* cassettes have been reversed relative to the Cis arrangement. The HO break within *leu2* on Chr XI is repaired by GC (using the *LEU2* donor on Chr III), and the HO break on Chr V is repaired by intrachromosomal SSA resulting in deletion of one of the *URA3* repeats. (D) Schematic of the Reverse-Trans strain (tNS2638) in which the positions of the 5’ truncated *ura3-HOcs-U2* cassette and the *LE-HOcs-URA3* cassette are switched relative to the Trans strain. Bottom panel indicates the repair outcome in which the *LE* end from the break on Chr V and the *U2* end from the break on Chr XI are repaired by GC (using the *LEU2* donor on Chr III), and the *ura3* end from the break on Chr XI and the *URA3* end from the break on Chr V are repaired by interchromosomal SSA resulting in deletion of one of the *URA3* repeats. Both repair events in this strain are also associated with a reciprocal translocation between chromosomes V and XI. Black arrows indicate the positions of the PCR primers used to study the kinetics of *LEU2* repair while the gray arrows indicate the position of primers used to examine the SSA repair.

Since the distance separating the strand-invaded DSB ends and their orientation are important signaling parameters during DSB repair, we wanted to test whether GC-mediated quick and efficient repair also required the two ends to come from the same break. Strain, YSJ379, referred to as Trans (Fig 1A) was designed to receive two DSBs, each on a different chromosome, created by galactose-inducible HO endonuclease. In this configuration one end from each of these DSBs can pair with a homologous donor on a third chromosome in a GC-compatible configuration. Specifically, the two ends of a *LEU2* gene (*LE* on Chr XI and *U2* on Chr V) can synapse with the *LEU2* donor on Chr III, and repair can be mediated by an ectopic recombination event–analogous to a gene conversion mediated by synthesis-dependent strand annealing (SDSA)–that results in a translocation between chromosomes V and XI. The other two ends–the *URA3* end from the break on Chr XI and the *ura3* end from the break on Chr V–can be repaired efficiently by interchromosomal single-strand annealing (SSA), also resulting in a chromosomal translocation and loss of one of the *URA3* repeats (Fig 1A). For comparison, we built another strain (YSJ357, designated Cis) (Fig 1B), which also harbors two HO-inducible breaks, one of which can be repaired by GC and the other by SSA. Here, the *LE* and *U2* ends that participate in the GC event originate from the same DSB on Chr V, while the second DSB on Chr XI, which is flanked by *ura3* and *URA3* sequences, is repaired by intrachromosomal SSA. We have shown previously and also confirm below that interchromosomal SSA is as efficient, and only slightly slower than intrachromosomal SSA [28]. Hence, using these two strains, we can compare the efficiency and kinetics of the repair events in which the *LE* and *U2* ends arise from a single break (Cis arrangement) or from two different breaks (Trans arrangement).

### Repair in Trans Is Kinetically Slower than Repair in Cis

**Fig 2:**
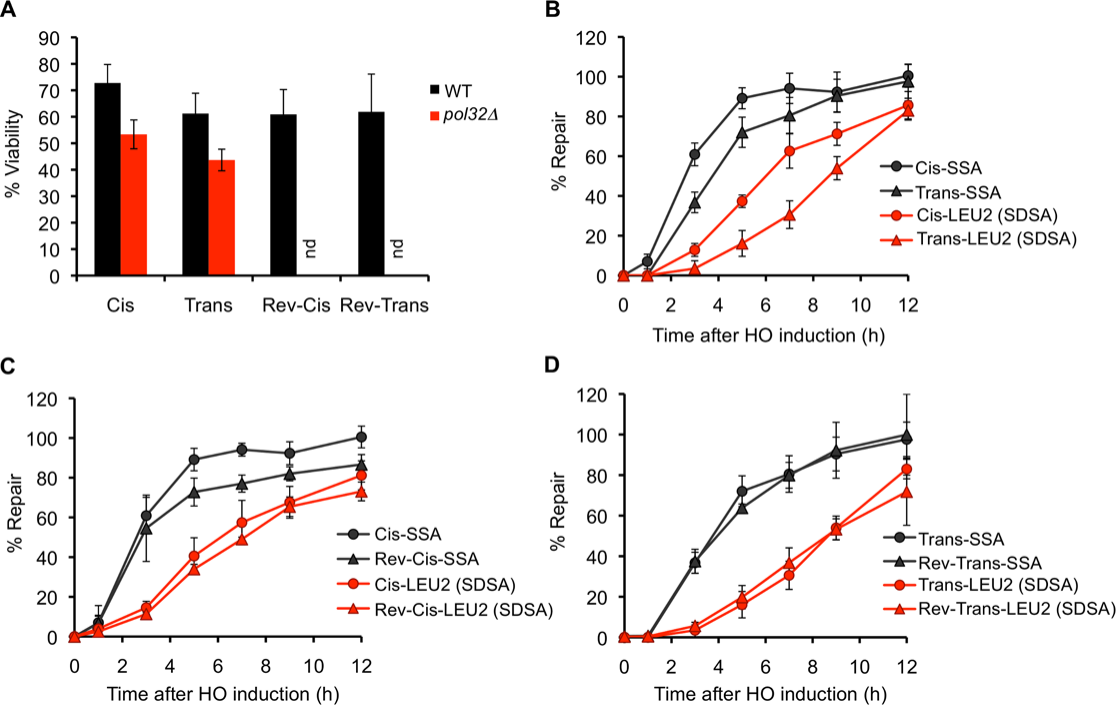
Repair in Trans is kinetically slower than repair in Cis. (A) Viabilities of the indicated strains (nd indicates not done). Data represent mean ± S.D. (n ≥ 6). (B-D) Kinetics of DSB repair in (B) Cis and Trans configurations, (C) Cis and Reverse-Cis configurations, and (D) Trans and Reverse-Trans configurations as determined by a quantitative PCR assay using primers shown schematically in Fig 1 and listed in S1 Table. The repair of *LE* and *U2* ends, which occurs predominantly by synthesis-dependent strand annealing, is indicated in the figure as SDSA, while the repair of the *URA3* and *ura3* ends, which occurs by single-strand annealing, is indicated as SSA. The amount of PCR product obtained from a repaired colony was used to make the standard curve for quantification. For Cis and Trans, data represent mean of a total of 6 PCR reactions from two independent time courses ± S.D. For Reverse-Cis and Reverse-Trans, data represent mean of three independent time courses ± S.D.

We first examined the efficiency of repair in these two strains by a viability assay and found that while ~73% of the cells were able to survive the breaks when the ends were in the Cis arrangement, ~61% of the cells survived the breaks in the Trans arrangement (Fig 2A). We then studied the kinetics of repair in these strains using a PCR assay with primer pairs as shown in Fig 1. We found that overall, the SSA product accumulated more rapidly than the GC product in both cases (Fig 2B); however, there was an hour-long delay in the appearance of the interchromosomal SSA product (Trans-SSA) relative to the intrachromosomal SSA product (Cis-SSA) (Fig 2B). This delay likely reflects the difference in the time it takes to find homology on the same chromosome (in Cis) as opposed to finding it on an altogether different chromosome (in Trans) [29,30], but may also be attributed to the competition in homology searching from the adjacent ends in the Trans configuration (see below). In contrast, the kinetics of *LEU2* repair were markedly different between Cis and Trans, with the Trans-*LEU2* product appearing at least 2 h after the appearance of the Cis product (Fig 2B), even though *LEU2* repair in both cases is an interchromosomal event. These differences in repair kinetics cannot be attributed to differential kinetics of HO break formation as our Southern assays show >90% cutting at the HO sites within an hour of HO endonuclease induction in both configurations (S1 Fig).

One of the differences between the Cis and Trans strains is that while the *U2* end originates from a break on Chr V in both cases, the *LE* end originates from a break on Chr XI in the Trans case as opposed to Chr V in the Cis case. Since the distal arm of Chr V does not contain any essential genes and can be lost without causing lethality, *U2*-mediated BIR events could also contribute to the overall efficiency of repair in the Cis arrangement (S2 Fig). However, in the Trans case, BIR will not yield a viable outcome; and this difference could explain the somewhat higher efficiency of repair in Cis relative to Trans. To address this possibility, we used a PCR-based approach to identify the proportion of colonies that had repaired the break in Cis by BIR (S2 Fig). We found that only ~2% of the repaired Cis colonies gave a product consistent with the BIR outcome indicating that the latter is not responsible for the higher efficiency of repair in Cis.

To further test whether the difference in efficiency and kinetics of *LEU2* repair between the Cis and Trans arrangements could be due to an effect of chromosomal context relating to the origin of the *LE* and *U2* ends in the two cases, we reversed the positions of the DSB cassettes in both configurations. We first built a Reverse-Cis strain, tNS2614, (Fig 1C) in which the positions of the *LE-HOcs-U2* and the 5’ truncated *ura3-HOcs-URA3* cassettes are reversed relative to the Cis strain such that the *LE* end is now present on Chr XI (just as in the Trans strain). We found that the overall efficiency of repair was somewhat reduced relative to the Cis strain, and only ~61% of the cells were able to survive the breaks in the Reverse-Cis configuration (Fig 2A). However, the kinetics of *LEU2* repair were quite comparable between the Cis and Reverse-Cis strains (Fig 2C) with the repair product appearing at least 2 h earlier in these configurations relative to the Trans arrangement. We also constructed an analogous Reverse-Trans strain, tNS2638, (Fig 1D) in which the positions of the *LE-HOcs-URA3* and the 5’ truncated *ura3-HOcs-U2* cassettes were reversed relative to the Trans strain. We found that the efficiency (Fig 2A) as well as the kinetics of repair (Fig 2D) were indistinguishable between the Trans and Reverse-Trans configurations.

Since the viability of the Reverse-Cis strain was comparable to that of the Trans and Reverse-Trans strains, we conclude that the configuration of the DSB ends *per se* does not affect the overall efficiency of repair. However, the slower kinetics of *LEU2* repair in the Trans and Reverse-Trans configurations is due to the involvement of DSB ends originating from two different breaks, as opposed to the Cis and Reverse-Cis cases where repair involves ends belonging to the same break.

**Fig 3:**
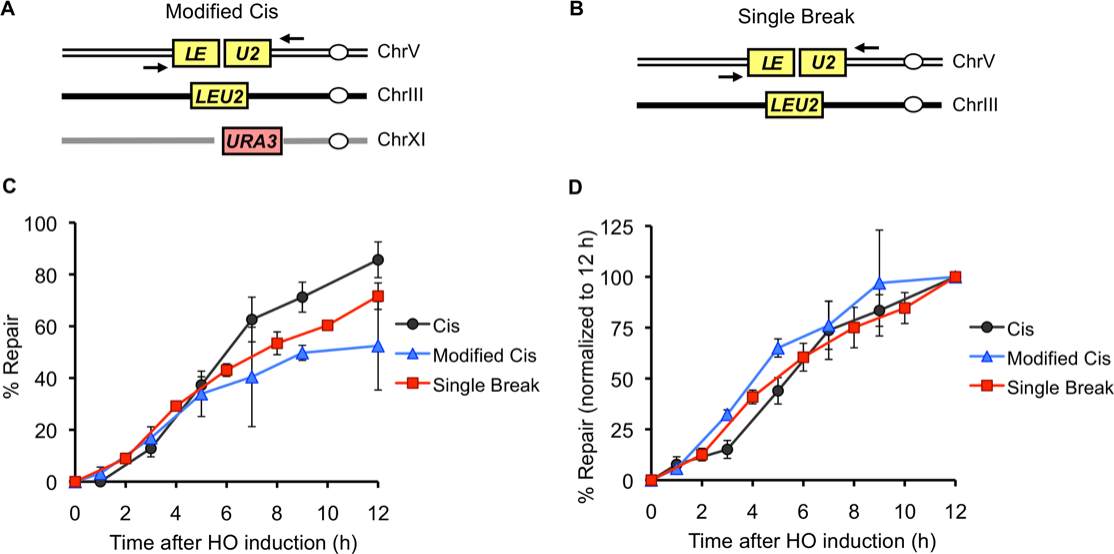
The kinetics of *LEU2* repair are independent of the repair outcome of another break. (A) Schematic representation of a modified Cis strain, tNS2607, which carries an *LE-HOcs-U2* cassette at the *can1* locus on Chr V, a *LEU2* donor on Chr III and an unrepairable *HOcs-URA3* break (instead of a *ura3-HOcs-URA3* cassette) on Chr XI. (B) Schematic representation of a strain which carries a *LE-HOcs-U2* cassette at the *can1* locus on Chr V and a *LEU2* donor on Chr III (YSJ119 [13]). This strain harbors a single HO break. (C) Kinetics of *LEU2* repair in the indicated strains, as determined by a quantitative PCR assay using primers shown schematically (black arrows) in Figs 1(B) and 3(A) and 3(B). The amount of PCR product obtained from a repaired colony was used to make the standard curve for quantification. (D) Data from (C) plotted after normalizing the amount of PCR product obtained at 12h time point for each strain to 100%. For Cis and Single Break, data represent mean of a total of 6 PCR reactions from two independent time courses ± S.D. For Modified Cis, data represent mean of three independent time courses ± S.D. The Single Break data shown in (C) has been published in [13].

Because there is an hour-long delay in the kinetics of *ura3* SSA repair in the Trans strain relative to the Cis strain (Fig 2B), we wondered if this delay could indirectly contribute to the slower kinetics of *LEU2* repair in this setting, perhaps by soaking up one or more repair factor(s). To address this possibility we studied the kinetics of *LEU2* repair in a modified Cis strain, which lacks one of the SSA substrates and therefore suffers an unrepairable break on Chr XI, in addition to the *LE-HOcs-U2* break on Chr V (Fig 3A). As a further control, we also compared the kinetics of *LEU2* repair in these strains with a strain that harbors just the single *LE-HOcs-U2* break on Chr V (Fig 3B, [13]). We found that although the overall repair efficiencies of these strains were different (Fig 3C), the kinetics of *LEU2* repair, when normalized to the amount of total product formed at 15 h, remained the same irrespective of the presence of an unrepairable break, or the lack of a second break altogether (Fig 3D). Overall, these data clearly demonstrate that GC-like repair involving ends from different breaks is kinetically slower than repair involving ends from the same break.

### Repair in Trans Does Not Appear To Be Mechanistically Different from Repair in Cis

The reduced efficiency and kinetics of *LEU2* repair in the Trans arrangement could be due a defect in signaling between the ends because they are unlinked to each other. Thus, even though the *LE* and *U2* ends will synapse next to each other in the correct orientation (with the *LEU2* donor), they might fail to generate a signal for quick repair because they don’t belong to the same break. In the absence of this signaling, the ends might slowly initiate two independent BIR events, whose products may subsequently anneal with each other giving rise to a GC/SDSA-like outcome [31,32]. To address this possibility, we deleted *POL32*, which is required for BIR but has only a modest effect on GC [14], to test if the kinetically slow Trans repair occurred by BIR rather than GC. Deleting *POL32* had a mild effect on the efficiency of repair in both Cis and Trans (Fig 2A), consistent with previous studies of its effect on GC [14]. The absence of a much greater defect in the Trans case strongly argues against the likelihood of repair in Trans being initiated by two independent BIR events. Hence, even though *LEU2* repair in Trans is kinetically slower, it does not appear to be mechanistically different from repair in Cis.

### Deleting Rad50, an End-Tethering Factor, Does Not Alter the Kinetics of Repair

**Fig 4:**
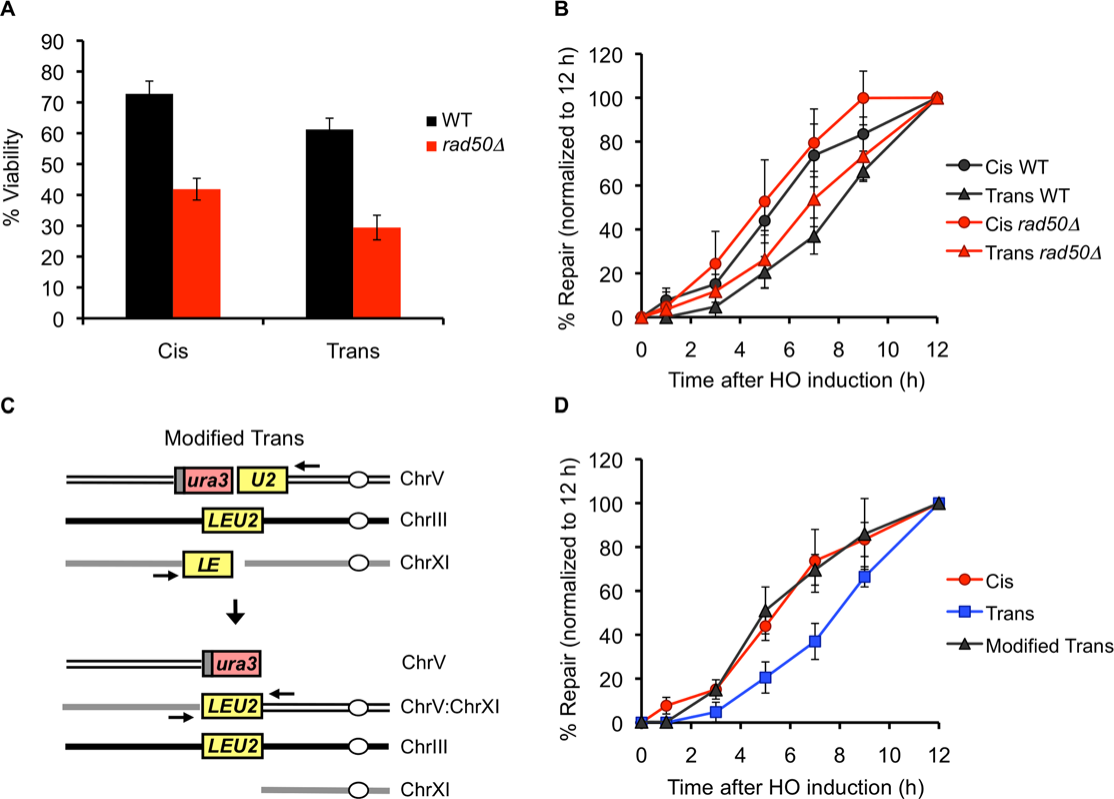
Eliminating the SSA substrate accelerates the kinetics of GC repair in Trans. (A) Viabilities of WT (black bars) and *rad50Δ* (red bars) Cis and Trans strains. Data represent mean ± S.D. (n ≥ 5). (B) Kinetics of *LEU2* repair in the indicated WT (black curves) and *rad50Δ* (red curves) strains, as determined by a quantitative PCR assay using primers shown schematically (black arrows) in Fig 1(A) and 1(B). The amount of PCR product obtained from a repaired colony was used to make the standard curve for quantification, and the data was plotted after normalizing the amount of product obtained at the 12 h time point to 100% in each case. For the repair kinetics in the WT background, data represent mean of a total of 6 PCR reactions from two independent time courses ± S.D. For the repair kinetics in the *rad50Δ* background, data represent mean of three independent time courses ± S.D. (C) Schematic representation of a modified Trans strain, tNS2628, which carries a 5’ truncated *ura3-HOcs-U2* cassette at the *can1* locus on Chr V, a *LEU2* donor on Chr III and an unrepairable *LE-HOcs* break (instead of an *LE-HOcs-URA3* cassette) on Chr XI. Bottom panel indicates the repair outcome in which the *LE* end from the break on Chr XI and the *U2* end from the break on Chr V are repaired by GC using the *LEU2* donor on Chr III. However, the other two ends of these breaks cannot be repaired due to the lack of a *URA3* substrate on the Chr XI. (D) Kinetics of *LEU2* repair in the indicated strains as determined by a quantitative PCR assay using primers shown schematically (black arrows) in Figs 1(A) and 1(B) and 4(C). The amount of PCR product obtained from a repaired colony was used to generate a standard curve for quantification, and the data was plotted after normalizing the amount of product obtained at the 12 h time point to 100% in each case. For Cis and Trans, data represent mean of a total of 6 PCR reactions from two independent time courses ± S.D. For the Modified Trans, data represent mean of three independent time courses ± S.D.

The MRX complex, Tel1, Sae2 γ-H2AX and Rad52 proteins have all been implicated in holding the ends of a single DSB together for up to several hours after break formation [24–27]. We wondered whether this end-tethering might be responsible for the quicker kinetics of repair in the Cis case. Synapse formation may happen more quickly and efficiently in the Cis strain where the *LE* and *U2* ends can travel together compared to the Trans strain where the *LE* and *U2* ends must independently seek out and pair with the *LEU2* donor. To examine the possible effect/s of end-tethering, we deleted *RAD50* to see if releasing the ends in the Cis arrangement would make them behave more like ends in the Trans arrangement, resulting in similar *LEU2* repair kinetics. We note that deleting *RAD50* or its partners *MRE11* and *XRS2* –in addition to reducing end-tethering [25,26]–also causes defects in both 5’ to 3’ resection of DSB ends and the DNA damage checkpoint [33–36], but those effects should be identical in our Cis and Trans arrangements and therefore, should not contribute toward equalizing repair in Cis and Trans. Deleting *RAD50* reduced the repair efficiency in both Cis and Trans to ~55% of their respective WT levels (Fig 4A). Although the kinetics of Cis and Trans repair in the absence of Rad50 looked rather similar at a first glance (S3 Fig), when we normalized the PCR assays to the total amount of product formed, we found that *rad50*Δ did not affect the overall kinetics of repair in either case (Fig 4B). We note that disruption of the MRX complex (as well as deletion of other tethering factors such as Sae2, γ-H2AX and Tel1) has been shown to eliminate end-tethering in only ~10–25% of the cells [25,26]; it is possible that this partial un-tethering of the DBS ends may not be sufficient to alter the repair kinetics in our assay.

### Eliminating the SSA Substrate Accelerates the Kinetics of GC Repair in Trans

In addition to facilitating the homology search by the DSB ends in Cis (as suggested above), DSB end-tethering might specifically impede repair in Trans, wherein the ability of *LE* and *U2* ends to locate their donor templates might be affected by the SSA event involving the *URA3* sequences on the other side of each DSB. For example, the *URA3* end from the break on Chr XI might drag its associated *LE* end to the *ura3* sequences on Chr V, thereby preventing *LE* from finding its homologous *LEU2* donor on Chr III. To address this possibility, we modified the Trans strain such that it no longer harbors the *URA3* SSA substrate on Chr XI (Fig 4C). Even though this strain does not generate viable repair outcomes, the *LE* end from Chr XI and *U2* end from Chr V can still repair by GC using the *LEU2* donor on Chr III, an event that can be monitored by PCR. We found that in the absence of a *URA3* substrate, the kinetics of *LEU2* repair in Trans were indistinguishable from those of Cis (Fig 4D). Hence, we conclude that in the original Trans strain, the *LE* and *U2* ends are inhibited from interacting with their donor sequences because of their association, across the “divide” of the DSB, with the *URA3* sequences, which are involved in a separate repair event. Conversely, the association of *URA3* sequences with both the *LE* and *U2* ends might be partially responsible for the slower kinetics of SSA repair in the Trans arrangement (Fig 2B).

### Adjacent Sequences Sharing Homologies with Other Loci Can Greatly Affect HR-Mediated Repair

**Fig 5:**
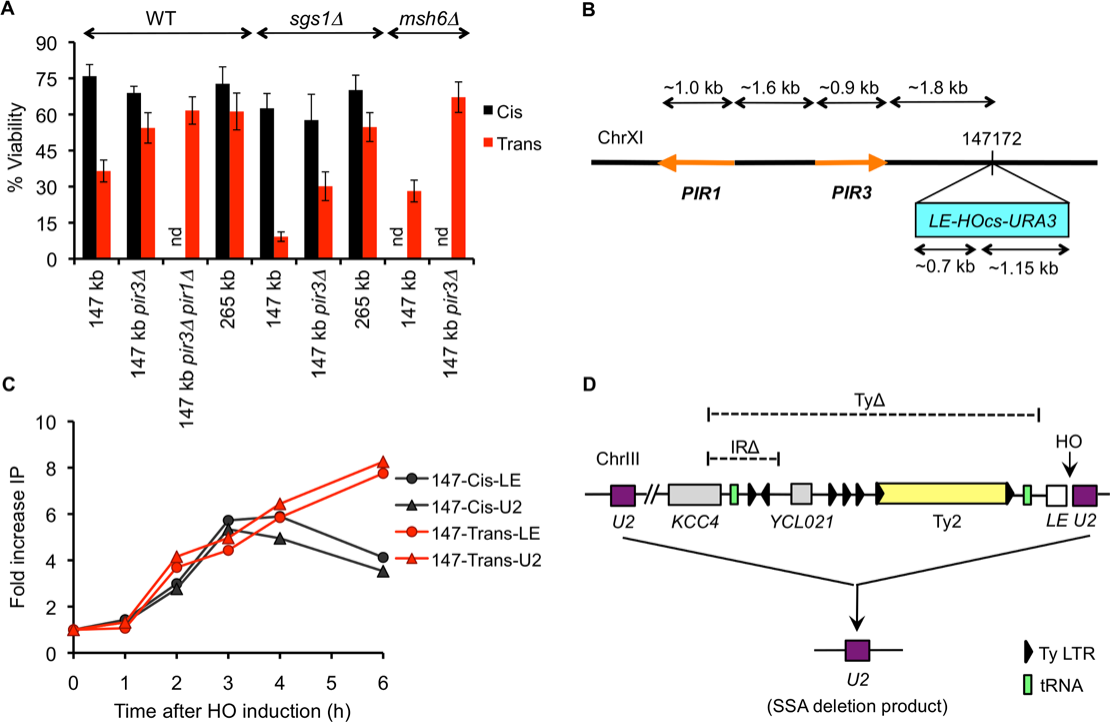
Adjacent *PIR* sequences interfere with GC repair in Trans. (A) Viabilities of the indicated WT, *sgs1Δ* and *msh6Δ* 147- and 265- Cis and Trans strains (nd indicates not done). Data represent mean ± S.D. (n ≥ 5). (B) Schematic representation of Chr XI features surrounding the site of insertion of the *LE-HOcs-URA3* cassette in the 147-Trans strain. The *LE-HOcs-URA3* cassette was inserted at position 147142 on the left arm of Chr XI. Orange lines represent the *PIR* genes and the arrowheads indicate their relative orientations. The distance of *PIR3* gene from *PIR1* and *LE-HOcs-URA3* cassette is indicated. The corresponding Cis strain contains a *NAT*-marked 5’ truncated *ura3-HOcs-URA3* cassette at position 147172. (C) Rad51 ChIP signal at the *LEU2* donor on Chr III representing the kinetics of strand-invasion by *LE* and *U2* ends in 147- Cis and Trans strains. Primers 300 bp and 200 bp upstream of the *LEU2* donor and 150 bp and 25 bp downstream of the *LEU2* donor were used to study the kinetics of strand-invasion by the *LE* and *U2* ends, respectively. (D) Schematic representation of the YMV80 SSA strain harboring an *HOcs* within the *leu2* gene at its endogenous locus, and a homologous *U2* sequence at the *his4* locus ~25 kb distal to the *LE-HOcs-U2*. Dotted lines indicate the regions spanning the Ty2 retrotransposon element, Ty1 LTRs (long terminal repeats) and tRNA genes that have been deleted in the TyΔ and IRΔ strains, respectively. TyΔ results in a net deletion of 4.7 kb. Figure not drawn to scale.

In addition to the Cis and Trans strains used in the above experiments in which the 5’ truncated *ura3-HOcs-URA3* cassette and the *LE-HOcs-URA3* cassette for Cis and Trans strains, respectively, were inserted ~265 kb from the left end of Chr XI (referred to hereafter as the 265- Cis and Trans strains), we had constructed another set of strains in which these cassettes were inserted distal to the *YKL162C* locus ~147 kb from the left end of Chr XI (referred to hereafter as the 147- Cis and Trans strains). While the repair efficiencies of the 265- and 147- Cis strains were comparable, the viability of the 147-Trans strain was only ~36% as opposed to the ~61% viability of the 265-Trans strain (Fig 5A). We speculated that the much reduced repair efficiency of the 147-Trans strain could be attributed to chromosomal context, wherein the neighboring sequences might interfere with repair. Indeed, a closer examination of the *YKL162C* locus (the site of insertion of the *LE-HOcs-URA3* cassette on chromosome XI) revealed that ~1.8 kb upstream of the *LE* sequences (and ~2.5 kb from the DSB end) lies the *PIR3* gene, which shares about 70% homology with two other genes–*PIR1* and *PIR2*. *PIR2* is present on Chr X, but *PIR1* lies ~1.6 kb further upstream of *PIR3* in an inverted orientation (Fig 5B). This arrangement of *PIR3* and *PIR1* plus the presence of two regions (77 and 81 bp) of perfect homology between these sequences raised the possibility that if 5’ to 3’ resection from the DSB end extends ~2.5 kb, it could promote recombination between *PIR3* and its homeologs; moreover, if resection extended ~6 kb, *PIR3* could fold back upon *PIR1* to form a stem-loop structure [37,38] and/or form SSA-mediated inter-chromatid dimers [38], both of which could preclude the *LE*-mediated repair in Trans. Therefore, it seemed possible that the *PIR3* sequences might interact with *PIR1* and/or *PIR2*, thereby interfering with *LEU2* repair in Trans.

To test whether *PIR3* sequences are indeed responsible for impeding *LEU2* repair in Trans, we re-inserted the *LE-HOcs-URA3* cassette at the same locus on Chr XI while deleting the adjacent *PIR3* sequences. An equivalent Cis strain was made by re-inserting the 5’ truncated *ura3-HOcs-URA3* cassette on Chr XI with a similar deletion of *PIR3*. In these *pir3Δ* strains, the viability of the Cis strain was ~69% and that of the Trans strain was ~54% (Fig 5A). Hence, while the *PIR3* deletion had no effect on repair in Cis, it significantly improved the efficiency of repair in Trans. Furthermore, double deletion of *PIR1* and *PIR3* increased the viability of the 147-Trans strain to ~61%, which was indistinguishable from that of the 265-Trans strain (Fig 5A).

It has been well established that Sgs1 plays a key role in preventing homeologous recombination [39–41]. We found that deletion of *SGS1* severely compromised repair in the 147-Trans arrangement (Fig 5A), presumably by further sequestering the *LE* end as a result of increased homeologous interactions between the *PIR3* gene and its homologues. When we deleted *PIR3* in the context of *sgs1Δ*, the efficiency of repair in 147-Trans increased from <10% in the *sgs1Δ* strain to ~30% in the *sgs1Δ pir3Δ* strain, which is still only ~50% of the *SGS1 pir3Δ* strain (Fig 5A). We suggest that in the *pir3*Δ strain, Sgs1 still limits homeologous interactions between *PIR1* and *PIR2* sequences. To investigate whether Sgs1 exerts its effect through the mismatch repair pathway, we deleted *MSH6* in the 147-Trans strain. Unlike *sgs1Δ*, deletion of Msh6 only mildly affected the efficiency of 147-Trans repair, and the latter was completely rescued by the deletion of *PIR3* (Fig 5A). Overall these results argue that interference from the neighboring *PIR3* and *PIR1* sequences is largely responsible for the much-reduced efficiency of repair in the 147-Trans strain. Surprisingly, strand-invasion of the *LEU2* donor by the *LE* and *U2* ends (as assessed by a Rad51 chromatin immunoprecipitation assay) appeared to occur with similar efficiency and kinetics in Cis and Trans (Fig 5C) suggesting that the *PIR* sequences may not affect synapse formation *per se*. Instead, this result argues that 5’ to 3’ resection continues even after the initial contact of the DSB end with its homologous donor sequences. A similar interpretation can be drawn from the data of Wu et al. [42] or Agmon et al. [29], who found that ectopic gene conversion could be followed by an intrachromosomal SSA event in which 5’ to 3’ resection continued into the flanking homologous region, past the sequences involved in GC.

Although the adjacent *PIR3* and *PIR1* sequences interfere with GC-mediated *LEU2* repair in the Trans strain, they do not affect the overall efficiency of repair in the 147-Cis strain (Fig 5A). Since the *PIR3* and *PIR1* sequences are present at the same distance away from the *ura3-HOcs-URA3* cassette in the 147-Cis strain as they are from the *LE-HOcs-URA3* cassette in the 147-Trans strain, the data from WT and *pir3Δ* 147-Cis strains imply that these *PIR* sequences do not interfere with intrachromosomal SSA. This difference is perhaps due to the much faster kinetics of SSA compared to GC, which may not allow enough time for resection to continue past the *PIR3* sequences before the completion of SSA.

### Effect of Competing Sequences on the Efficiency of SSA

The results presented above as well as previous studies have suggested that sequences distant from the DSB can participate in competing recombination events thereby interfering with recombination of sequences closer to the break [43]. In another assay system, we found that the presence of a nearby copy of a family of dispersed, repeated sequences impaired the efficiency of an HO-induced SSA event. We have previously described strain YMV80, in which an HO-induced DSB is created within the *leu2* gene (at its original location on Chr III). This break is repaired by SSA between the *U2* sequence next to the cleavage site and a ~1 kb *U2* sequence inserted at the *his4* locus ~25 kb distal to the *HOcs*, resulting in a 25 kb deletion of the nonessential sequences between the *U2* repeats [30]. Repair takes approximately 6 h, consistent with the amount of time required to resect 25 kb of DNA at ~4 kb/h rate of resection. Distal to the *LEU2* locus and between the flanking *U2* segments lies a complete Ty2 retrotransposon element plus several LTRs (long terminal repeats), as well as two tRNA genes (Fig 5D). In the presence of these repeated sequences, the efficiency of *U2*-mediated SSA repair, as measured by a viability assay, was found to be 76±10%. However, when these intervening repeated sequences were deleted (Fig 5D, TyΔ), the repair efficiency increased to 100±15%.

To explore this further, we also deleted a short region to the left of Ty2 containing an inverted repeat (IR) of the Ty1 LTRs and a tRNA gene. However, this deletion (Fig 5D, IRΔ) did not affect the efficiency of repair (81±3%) relative to the parent strain. These observations suggest that resection into the Ty2 element allows it to recombine with some of the other ~30 copies of Ty elements and even more LTRs dispersed throughout the genome, most of which would yield inviable outcomes. Thus, removing these potentially competing sequences greatly enhances repair by SSA and provides another example of impediment of DSB repair by nearby repeated sequences.

## Discussion

We have previously shown that a Recombination Execution Checkpoint regulates the choice of the HR pathway based on whether the two ends of a DSB strand-invade closely positioned and correctly oriented homologous donor sequences [13]. We reported that increasing the distance between the homologous donors (while the site of DSB induction was kept constant) resulted in REC-mediated shift from GC to BIR mode of repair. Here we tested whether REC can also detect the origin of the ends that are engaged in repair, since repair involving ends from different breaks can produce deleterious chromosomal translocations. We therefore examined the effect of separating the origin of the DSB ends while the donors were kept constant and positioned next to each other in the correct GC-compatible configuration. We found that involvement of ends from two different breaks did not compromise the overall efficiency of repair (Fig 2A). However, it slowed down the kinetics of repair quite dramatically as we observed a ~2 h delay in the appearance of product in the Trans and Reverse-Trans case relative to the Cis and Reverse-Cis configurations (Fig 2B–2D). Contrary to our expectation, we found that just like the Cis case, repair in Trans was largely Pol32-independent (Fig 2A) indicating that although it is kinetically slower, repair in Trans still proceeds via GC. Hence, the REC cannot distinguish between the origins of the synapsed ends and does not specifically restrict GC-mediated repair of ends belonging to two different breaks. This finding argues that the while physical distance separating the synapsed ends is a critical parameter that governs the REC-mediated switch between GC and BIR, if the DSB ends are synapsed close to each other (in the correct orientation) on the donor template, the GC requirement is fulfilled and the origin of the ends has no relevance. This observation supports our previous hypothesis that the REC acts after the strand-invasion step whereby it prevents loading of the GC machinery to single-end invasions, or if the ends have invaded far away from each other, or in the wrong orientation. We conclude that when two DSB ends, irrespective of their origin, are synapsed close to each other in the correct orientation, the REC allows the GC machinery to carry out repair before BIR can come into play.

However, we did observe slower kinetics of repair in Trans. Our data indicate that this delay is attributable to DSB end-tethering. Using fluorescently tagged arrays, it has previously been shown that DSB ends remain tethered together up to several hours after induction of an irreparable break in a vast majority of the cells [24–27]. This tethering is partially dependent upon the MRX complex, as well as Sae2, Tel1 and Rad52 proteins. While end-tethering has been shown to play a very important role in preventing NHEJ-mediated chromosomal translocations, its role in GC has remained elusive because these proteins are pleiotropic and affect many other aspects of repair as well, such as G_2_/M checkpoint activation, 5’ to 3’ end resection and DSB-induced sister-chromatid cohesion [33–36]. Therefore, it is difficult to ascertain whether or not a repair defect observed upon disruption of these proteins is attributable to the loss of end-tethering *per se*. We reasoned that our Trans strain would overcome this problem because the ends that are involved in GC-mediated repair don’t belong to the same break, and therefore, would not be tethered to each other (although they would presumably be tethered to the *URA3* sequences on the opposite sides of the DSBs). Since the cells are able to survive a break in the Trans and Reverse-Trans configuration as efficiently as a break in the Reverse-Cis configuration (in which the ends involved in repair should be tethered) (Fig 2A), DSB end-tethering seems to be largely dispensable for the successful completion of GC-mediated repair. Nevertheless, the delay in the kinetics of repair in Trans and Reverse-Trans might reflect the lack of association between *LE* and *U2* ends, which would have to independently seek out their homologous sequences, as opposed to repair in the Cis and Reverse-Cis configurations where the *LE* and *U2* ends would be tethered together and might therefore engage in a coordinated search for their homologous partners. Alternatively, the slower kinetics of *LEU2* repair in Trans and Reverse-Trans could be an indirect effect of tethering of the *LE* and *U2* ends to the corresponding *URA3* sequences on the other side of the DSBs, which are involved in a separate SSA repair event and might therefore drag the associated *LE* and *U2* ends away from their homologous donor. While *RAD50* deletion reduced the efficiency of both Cis and Trans repair, it did not alter their relative kinetics (Fig 4A and 4B). We suspect that this is due to only partial un-tethering of the DSB ends in the absence of Rad50 [25,26]. Indeed, when we removed the influence of association to the other end by eliminating the SSA event in the Trans setting, the kinetics of Trans GC became as rapid as in the Cis case (Fig 4D). These data argue that in the original Trans strain, the *URA3* sequences that are involved in SSA-mediated repair, potentially drag their associated *LE* and *U2* ends away from the *LEU2* donor on Chr III. This, in turn, would compromise the probability of *LE* and *U2* ends simultaneously engaging the *LEU2* donor, resulting in a smaller proportion of cells initiating GC-mediated repair at any given time. Overall these results suggest that end-tethering may play a substantial role in preventing chromosomal translocations that could arise from GC repair involving ends from different breaks. This might become particularly important if a break occurs within a repeated sequence such that the ends could either travel together and repair using donors present in the vicinity of each other or engage in separate GC events involving unlinked donors.

We did not see a difference in the kinetics or efficiency of strand-invasion of the *LEU2* donor between our 147- Cis and Trans strains (Fig 5C); however, ChIP gives a bulk estimate and does not tell us the proportion of cells in which both ends are simultaneously synapsed with the homologous donor–a parameter that seems to play a critical role in regulating the initiation of repair synthesis.

We also found that sequences adjacent to those engaged in an HR event have a profound effect on the outcome. This is most evident in the case of the 147-Trans strain in which the adjacent *PIR3* sequences greatly interfere with GC-mediated repair (Fig 5A). If 5’ to 3’ resection is extensive before (or maybe even after) *LE* succeeds in finding its homologous donor, single-stranded DNA at *PIR3* may fold back upon *PIR1* (which lies just ~1.6 kb upstream of *PIR3* in the inverted orientation) and/or it may engage in some other homeologous interactions with *PIR1* or *PIR2*, with which it shares ~70% homology, thereby driving the *LE* end away from its GC template. These *PIR3* interactions can at least partially be disrupted by Sgs1 (Fig 5A), which has previously been shown to prevent gross chromosomal rearrangements (GCRs) between homeologous sequences [39,41].

Because Sgs1 has roles in processes other than mismatch repair, we also deleted *MSH6* to impair heteroduplex rejection during interactions between *PIR3* and *PIR1*/*PIR2*. The much weaker effect of *msh6Δ* on the viability of the 147-Trans strain may be due to its redundancy with Msh2-Msh3 in heteroduplex rejection. We did not test *msh2Δ* and *msh3Δ* mutants due to their essential role in removal of the non-homologous tails during SSA. Moreover, Sgs1 has been similarly reported to discourage spontaneous interchromosomal recombination among a set of highly diverged genes (*CAN1*, *LYP1* and *ALP1*) independently of *MSH6* and *MSH2*. The overall divergence of *CAN1* and *LYP1* or *ALP1* is very high (64% sequence identity) and many recombination events occurred in a stretch of DNA that shared ~74% sequence identity, which is comparable to the sequence similarity between *PIR* genes. Hence, we conclude that homeologous interactions between *PIR* genes are responsible for impeding *LEU2* repair in the 147-Trans strain.

Nearby repeated sequences also affect long-range SSA. Unlike GC, SSA will delete the repeated sequences in the intervening region in surviving cells; yet they still influence the outcome. It is possible to envision several possible modes of impairment. The two ends of the DSB may be tethered so that when the Ty sequences engage another repeated sequence, the *U2* sequences may be dragged along, thus restricting its interaction with the second *U2*. It is also possible that the Ty recombines with another Ty and generates an inviable chromosomal configuration. Alternatively, the repeated sequences may generate an intermediate that prevents DNA resection or in some manner interferes with SSA. We note that deletion of the Ty element results in net deletion of ~4.7 kb in the 25-kb region between the *U2* repeats (see Methods). We think it is unlikely that this change in the separation of the *U2* sequences could be responsible for the increase in repair efficiency from ~76% to ~100%, because increasing the distance between the *U2* repeats from ~25 kb to ~30 kb was not found to reduce the efficiency of SSA [30].

The effect of chromosomal context may also explain the difference in repair between the Cis and Reverse-Cis strains. Recent studies by Agmon et al. [44], emphasize that sequences at some chromosome locations recombine more readily with other sequences located at equivalent sites in the yeast nucleus. Interference from Ty elements has been seen before [38,45,46] and it has been shown that sequences further away from a break can drive repair as efficiently as the sequences immediately flanking the DSB [43]. Such interference from neighboring sequences can pose a big challenge for HR-mediated repair, especially in mammalian cells, which possess repeated sequences throughout their genome.

## Methods

### Strains and Plasmids

All strains were derived from YSJ119 (*ho hml*Δ::*ADE1 mata*Δ::*hisG hmr*Δ::*ADE1 leu2*::*KAN ade3*::*GAL*::*HO ade1 lys5 ura3-52 trp1 can1*::*LE-HOcs-U2 LEU2 at position 41400* of Chr III [13]. YSJ159 was constructed by replacing the endogenous *ura3-52* in YSJ119 with *HPH*, and YSJ352 was obtained by replacing *leu2*::*KAN* in YSJ159 with *leu2*::*LYS5*. The Cis strain YSJ357 was constructed by inserting a 5’ truncated *ura3-HOcs-URA3* cassette (from pSJ20) at position 265602 on Chr XI in YSJ352. The Trans strain YSJ379 was constructed in several steps. First, the *LE* portion of the *LE-HOcs-U2* cassette at the *can1* locus on Chr V in YSJ159 was replaced with a *NAT*-5’ truncated *ura3* sequence (from pSJ18) to obtain YSJ165. Next, *leu2*::*KAN* in YSJ165 was replaced with *leu2*::*LYS5* to obtain strain YSJ353. Finally, an *LE-HOcs-URA3* cassette (from pSJ17) was inserted at position 265602 on Chr XI in YSJ353 to obtain YSJ379. Plasmid pSJ18 was constructed by inserting an 800 bp long 5’ truncated *ura3* sequence between the SacI and SpeI sites in pAG25. pSJ20 was constructed by inserting an *HOcs-URA3* fragment (from pSJ17) at the SpeI site in pSJ18. pSJ17 was obtained by inserting *URA3* at the AgeI site in pJH1386 (which carries the XhoI-SalI *leu2* fragment containing the 117 bp *HOcs* at the Asp718 site). The Reverse-Cis strain (tNS2614) was derived from YSJ352 in several steps. First, the *can1*::*LE-HOcs-U2* cassette on Chr V was replaced with the 5’ truncated *ura3-HOcs-URA3* cassette (from pSJ20) to obtain tNS2563. A *NAT-LE-HOcs-U2* cassette derived from plasmid pNSU283-3 was inserted at position 265602 between *BUD2* and *MBR1* on Chr XI in tNS2563 to obtain tNS2605. Subsequently, the *NAT* marker was swapped with *TRP1*, and the *trp1* allele on Chr IV was replaced with *trp1*::*NAT* to create the Reverse-Cis strain. Plasmid pNSU283-3 was constructed by inserting *LE-HOcs-U2* into pAG25 [47] between the EcoRI and SpeI sites and chr XI sequences were added to each side of the *LE-HOcs-U2* cassette using the Gibson Assembly method [48]. The reverse-trans strain, tNS2638, was constructed by first PCR amplifying the *NAT*-*ura3*-*HOcs*-*U2* cassette from YSJ379 and inserting it at position 265602 between *BUD2* and *MBR1* on Chr XI in YSJ352. The second cassette, *LE*-*HOcs*-*URA3*, was amplified from YSJ379 and inserted at the *can1* locus on Chr V. The modified Cis strain, tNS2607, was derived from YSJ159 by inserting the *HOcs-URA3* cassette at position 165957 between *MBR1* and *BUD2* on Chr XI. To construct the modified Trans strain, tNS2628, we replaced the *NAT-ura3* portion of the *NAT-ura3-HOcs-U2* cassette on ChrV in YSJ379 with *TRP1* and subsequently replaced the *trp1* allele on Chr IV with *trp1*::*NAT*. The 147-Cis strain (YSJ176) was derived from YSJ159 by inserting a 5’ truncated *ura3-HOcs-URA3* cassette (from pSJ20) at position 147172 on ChrXI. The 147-Trans strain (YSJ175) was constructed by inserting an *LE-HOcs-URA3* cassette (EcoRI-BamHI fragment from pSJ19) at position 147172 on ChrXI in YSJ165. pSJ19 was constructed by inserting the *LE-HOcs-URA3* fragment (from pSJ17) at the HindIII site in pSJ16 (pBR322 carrying ChrXI sequences from positions 146537 to 147378 between EcoRI and BamHI sites). YSJ194 and YSJ195 were derived from YSJ175 and YSJ176, respectively, by replacing the *leu2*::*KAN* cassette with *LYS5*. YSJ363 (*pir3Δ* version of the 147-Cis strain) was made by inserting the 5’ truncated *ura3-HOcs-URA3* cassette (from pSJ20) between positions 144273 and 147176 on ChrXI in YSJ352. YSJ377 (*pir3Δ* version of the 147-Trans strain) was made by inserting the *LE-HOcs-URA3* cassette (from pSJ17) between positions 144273 and 147192 on ChrXI in YSJ353. tNS2646 (*pir1*Δ *pir3*Δ version of the 147-Trans strain) was made by inserting the *LE-HOcs-URA3* cassette (from pNSU290) between positions 141790 and 145384 on ChrXI in YSJ353. *pol32Δ*, *rad50*, and *sgs1Δ* strains were made by the standard PCR-based gene disruption method to obtain strains YSJ398 (YSJ379 *pol32Δ*), YSJ399 (YSJ357 *pol32Δ*), YSJ382 (YSJ379 *rad50Δ*), YSJ383 (YSJ357 *rad50Δ*), YSJ226 (YSJ194 *sgs1Δ*), YSJ227 (YSJ195 *sgs1Δ*), YSJ384 (YSJ377 *sgs1Δ*) and YSJ385 (YSJ363 *sgs1Δ*). The *msh6Δ* mutants were made by oligo-directed transplacement to create strains tNS2648 (YSJ194 *msh6Δ*) and tNS2650 (YSJ377 *msh6Δ*). YMV80 has been described before [30]. We first deleted the sequences between *KCC4* and *LE-HOcs-U2* in the YMV80 parent strain, YFP17 [30], using plasmid pNSU262 to obtain strain tNS2326. pNSU262 was derived from pAG32 [47] and harbors sequences flanking *KCC4* and *LEU2* to target a 8.8 kb deletion of Chr III encompassing the Ty element. Transplacement of pNSU262 results in addition of 4.1 kb of vector sequences resulting in a net deletion of 4.7 kb. A *TRP1*-*U2* was then inserted at the at the *his4* locus in this strain to create the strain tNS2333. tNS2427, possessing the deletion of the inverted repeat sequence, was obtained by oligo-directed transplacement of a PCR product using pNSU262 (*kcc4*-*HPH*) as the template and a target sequence located to the left of *YCL021W-A*.

### Viability Measurements

Yeast cells were grown in YEP containing 2% raffinose to a density of ~1x10^7^ cells/ml. Equal volumes of appropriate dilutions were plated on YEP containing 2% galacotse (YEPGal; to induce the HO break) and YEP containing 2% dextrose (YEPD; no DSB control). Viability was determined from the ratio of CFUs able to survive the break (number of colonies that grew on YEPGal) to the total number of CFUs plated (number of colonies that appeared on YEPD). Data represent mean ± S.D. (n ≥ 5), and p values were calculated using the Student’s *t*-test.

### HO Induction for Kinetic Analysis of DSB Repair

Yeast cells were grown in YEP containing 2% raffinose to a density of ~1x10^7^ cells/ml and HO endonuclease was induced by adding galactose to a final concentration of 2%. Samples were collected for DNA analysis just prior to and at different time points following addition of galactose, as described before [49].

### PCR-Based Primer Extension Assay

Equal amounts of genomic DNA isolated from samples collected at different time points were PCR-amplified (primer pairs are listed in S1 Table) within linear range as described before [50]. A pair of primers 300 bp upstream of *ura3* and 700 bp (for Cis and Trans) or 256 bp (for Reverse-Cis and Reverse-Trans) downstream of *URA3* was used to analyze the *URA3* repair kinetics. A primer pair with one primer downstream of *HOcs-U2* and another primer 150 bp (for Cis) or 650 bp (for Trans) upstream of the *LE-HOcs* sequence was used to analyze the kinetics of *LEU2* repair. For the Reverse-Cis strain the primer pairs were located 22 bp upstream of *LE-HOcs* and 100 bp downstream of *U2-HOcs*, and for the Reverse-Trans strain, the primers were located 150 bp upstream of *LE-HOcs* and 100 bp downstream of *U2-HOcs*. The PCR reactions were run on agarose gels and the repair product was quantified using Bio-Rad Quantity One software. For the Cis and Reverse-Cis strains, the primer pairs used to study *LEU2* and *URA3* repair give bands at 0 h time points as well; however, those bands are bigger than the ones obtained after repair (owing to presence of the 117 bp HOcs and the HOcs plus an additional *URA3* repeat, respectively) and were not used in our analysis. PCR signal from an independent locus (*SLX4*) was used to normalize for input DNA. The ratio of test and reference signals obtained from the DNA of a repaired colony was set to 100%.

### Colony PCR

A small quantity of cells taken from colonies growing on YEPGal (from the viability assay described above) were heated at 100°C for 10 minutes and the lysates were amplified with primers listed in S2 Table to assay for the loss of the distal arm of Chr V and appearance of the BIR product (as diagramed in S2 Fig).

### ChIP Assay

Rad51 ChIPs were performed as described [7]. The IP signal from the donor locus was normalized to the IP signal from the *CEN8* locus, which was immunoprecipitated using anti-Mif2 antibody.

## Supporting Information

S1 Fig Southern analysis of *LEU2* repair in Cis and Trans configurations. (A) Schematic representation of SphI restriction sites (vertical lines) in the indicated parent strains and their repair outcomes. (B) Southern analysis of *LEU2* repair in the indicated strains as a function of time following induction of the HO endonuclease. DNA isolated from cells harvested at different time points before and after HO induction was digested with SphI and probed with a *LEU2* probe. The *LEU2* donor on Chr III gives a 6.6 kb band in both configurations. In the Trans strain, the Chr V substrate (5.3 kb) and the Chr XI substrate (3.5kb) generate 4.1 kb and 1.5 kb bands, respectively, upon HO cleavage while the repaired product yields a 5.6 kb band. In the Cis strain, the Chr V substrate (8.3kb) is cut by the HO endonuclease to give bands that co-migrate at 4.1 kb, and the repaired product generates an 8.2 kb band. In the Cis configuration, the product band is only ~100 bps shorter than the substrate band owing to the loss of 117 bps HO cut site.
(PDF)

S2 Fig Scheme for detection of BIR-mediated repair in the Cis configuration. Upper panel is a schematic representation of the Cis strain and the lower panel indicates the *U2*-mediated BIR outcome. Proportion of colonies that repaired the break on Chr V by BIR was determined by colony PCRs using primers p1 and p2 (to assay for the loss of the distal arm of Chr V), and primers p3 and p4 (to assay for appearance of the BIR product) as a result of a non-reciprocal translocation between Chr V and Chr III. A total of 87 colonies were analyzed. Sequences of primers p1-p4 are listed in S2 Table.
(PDF)

S3 Fig Kinetics of *LEU2* repair in WT and *rad50Δ* Cis and Trans strains. Raw data is shown (not normalized to the amount of product obtained at the last time point).
(PDF)

S1 Table List of oligonucleotides used for the primer extension assays. (PDF)

S2 Table List of oligonucleotides used for the colony PCR assays. (PDF)
