# Supplementary figures and images for: Role of Double-Strand Break End-Tethering during Gene Conversion in Saccharomyces cerevisiae
Source: PLoS Genet. 2016 Apr 13;12(4):e1005976. doi: 10.1371/journal.pgen.1005976 (PMC4830573; doi:10.1371/journal.pgen.1005976)

**A**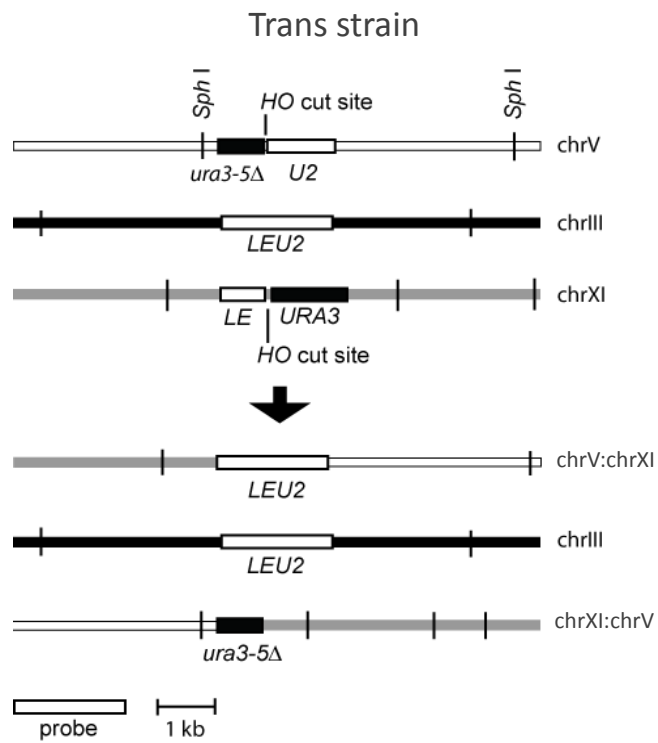**Cis strain**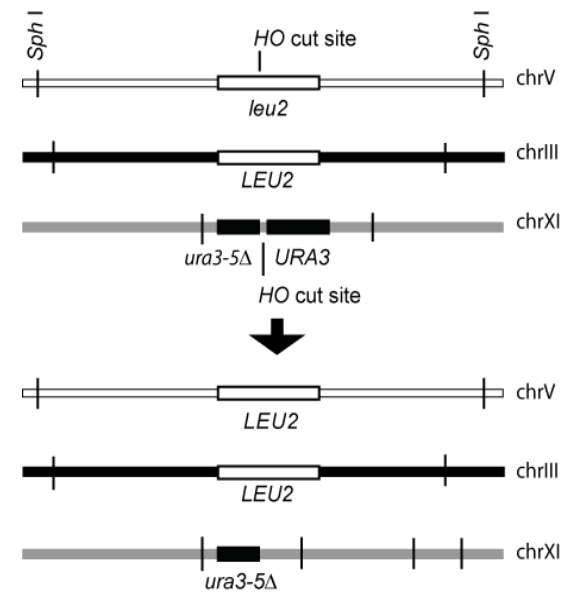**B**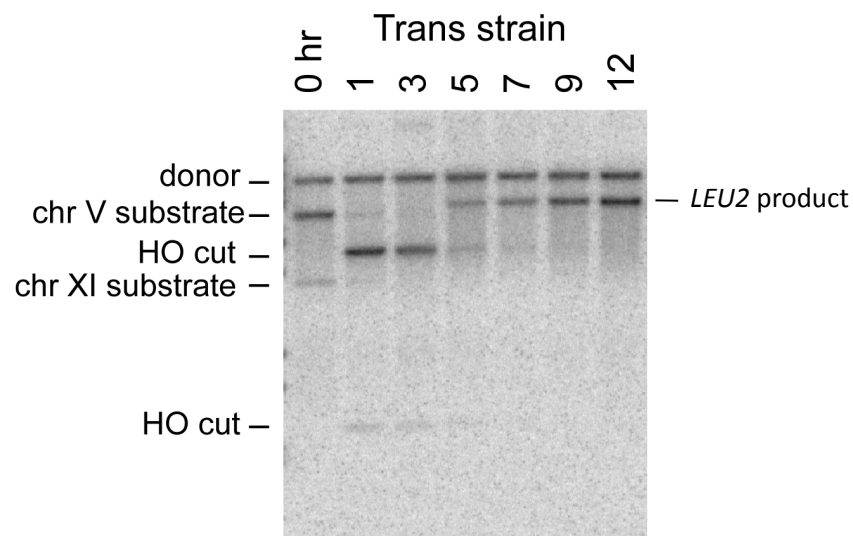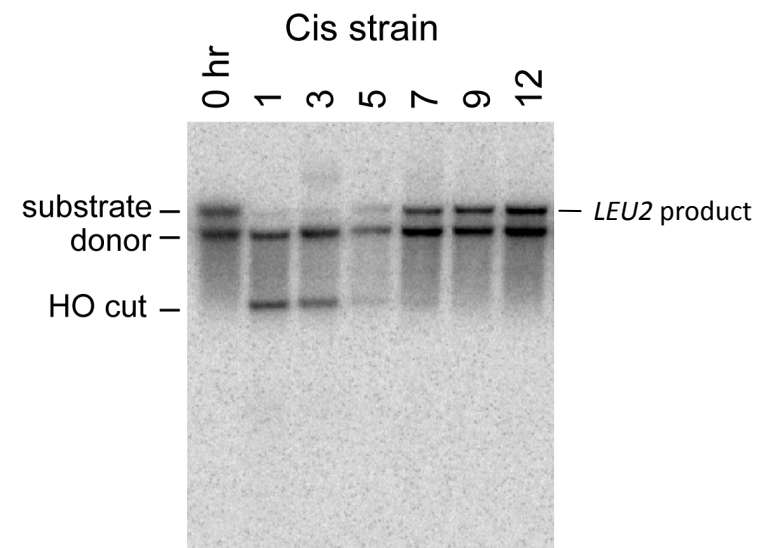

Supplement: S1 Fig — (A) Schematic representation of SphI restriction sites (vertical lines) in the indicated parent strains and their repair outcomes. (B) Southern analysis of LEU2 repair in the indicated strains as a function of time following induction of the HO endonuclease. DNA isolated from cells harvested at different time points before and after HO induction was digested with SphI and probed with a LEU2 probe. The LEU2 donor on Chr III gives a 6.6 kb band in both configurations. In the Trans strain, the Chr V substrate (5.3 kb) and the Chr XI substrate (3.5kb) generate 4.1 kb and 1.5 kb bands, respectively, upon HO cleavage while the repaired product yields a 5.6 kb band. In the Cis strain, the Chr V substrate (8.3kb) is cut by the HO endonuclease to give bands that co-migrate at 4.1 kb, and the repaired product generates an 8.2 kb band. In the Cis configuration, the product band is only ~100 bps shorter than the substrate band owing to the loss of 117 bps HO cut site. (PDF) [file pgen.1005976.s001.pdf]

Supplementary Figure 2

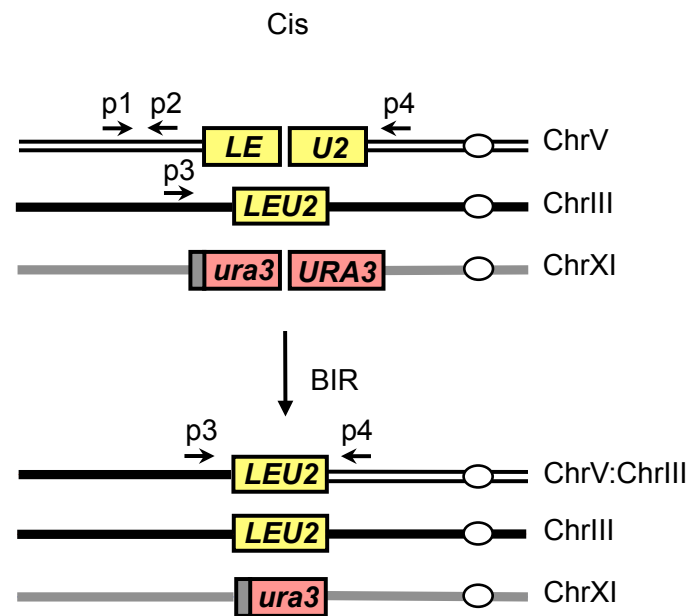

Supplement: S2 Fig — Upper panel is a schematic representation of the Cis strain and the lower panel indicates the U2-mediated BIR outcome. Proportion of colonies that repaired the break on Chr V by BIR was determined by colony PCRs using primers p1 and p2 (to assay for the loss of the distal arm of Chr V), and primers p3 and p4 (to assay for appearance of the BIR product) as a result of a non-reciprocal translocation between Chr V and Chr III. A total of 87 colonies were analyzed. Sequences of primers p1-p4 are listed in S2 Table. (PDF) [file pgen.1005976.s002.pdf]

Supplementary Figure 3

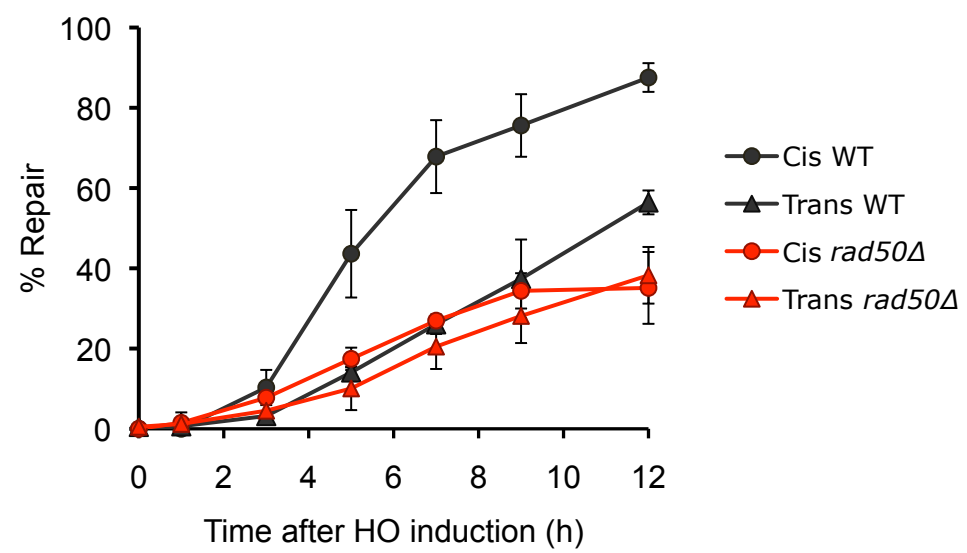

Supplement: S3 Fig — Raw data is shown (not normalized to the amount of product obtained at the last time point). (PDF) [file pgen.1005976.s003.pdf]
