# Supplementary material for: Role of Double-Strand Break End-Tethering during Gene Conversion in Saccharomyces cerevisiae
Source: PLoS Genet. 2016 Apr 13;12(4):e1005976. doi: 10.1371/journal.pgen.1005976 (PMC4830573; doi:10.1371/journal.pgen.1005976)
Supplement: S1 Table — (PDF) [file pgen.1005976.s004.pdf]

LEU2 repair

Cis	TGAGAATGCGAAATGGCGTGG<br>GAAAGAAGAGTGGTTGCGAACAGAG
Trans	AGCCAGTACAACAGCAACAG<br>GAAAGAAGAGTGGTTGCGAACAGAG
Rev-Cis	AGTATTGTTTGTGCACTTGCCT<br>TAGCGGGTTGTACTATCAGCAG
Rev-Trans	TGAGAATGCGAAATGGCGTGG<br>TAGCGGGTTGTACTATCAGCAG

URA3 repair (SSA)

Cis	TGTGAATGCTGGTCGCTATACT<br>GGAGCAAGAGTCATGTTTACTCTATAATAGG
Trans	TGTGAATGCTGGTCGCTATACT<br>GGAGCAAGAGTCATGTTTACTCTATAATAGG
Rev-Cis	TGAGAATGCGAAATGGCGTGG<br>CGAGGAAGACGATAAGGTTAAGATAAG
Rev-Trans	TGTGAATGCTGGTCGCTATACT<br>GAGTGGTTGCGAACAGAGTAAA

Reference locus (*SLX4)*

All strains	GAGGGTGGTGTGATCTTTAACC<br>ACGATCGAACAGCTTTGAATGA
