# Supplementary material for: Role of Double-Strand Break End-Tethering during Gene Conversion in Saccharomyces cerevisiae
Source: PLoS Genet. 2016 Apr 13;12(4):e1005976. doi: 10.1371/journal.pgen.1005976 (PMC4830573; doi:10.1371/journal.pgen.1005976)
Supplement: S2 Table — (PDF) [file pgen.1005976.s005.pdf]

p1	TCGGGTGTGTATTGGTTCCATTCA
p2	TGAATGTAAATGGAGAACCGTACGC
p3	GGGAAGGTGGGACAAAACTCAT
p4	GAAAGAAGAGTGGTTGCGAACAGAG
